# The use of youth healthcare services and its association with health-related quality of life, physical and mental health and over-the-counter analgesics use in 13–19-year-old adolescents: a cross-sectional study

**DOI:** 10.1186/s12889-023-17544-0

**Published:** 2024-01-05

**Authors:** Eva-Grethe Befus, Eirin Mølland, Sølvi Helseth, Milada Hagen, Tonje Holte Stea, Sandra Nolte, Kristin Haraldstad

**Affiliations:** 1 Faculty of Health and Sport Sciences, University of Agder, P.O. Box 422, Kristiansand, 4604 Norway; 2 Department of Economics and Finance, School of Business and Law, University of Agder, P.O. Box 422, Kristiansand, 4604 Norway; 3 Faculty of Health, Oslo Metropolitan University, St. Olavs plass, P.O. Box 4, Oslo, 0130 Norway; 4 Melbourne Health Economics, Centre for Health Policy, The University of Melbourne, Melbourne, VIC Australia; 5 School of Health Sciences, Swinburne University of Technology, Melbourne, VIC Australia

**Keywords:** Youth healthcare services, Public health nurse, Youth health centre, Health-related quality of life, Mental health, Pain, Headache, OTC analgesics, Immigrant, Adolescents

## Abstract

**Background:**

Youth healthcare services in Norway include a public health nurse (PHN) at school and local youth health centres (YHCs). They provide health services for all adolescents free of charge, focusing on health promotion and disease prevention. The present study aimed to assess possible associations between health-related quality of life (HRQoL), physical and mental health, over-the-counter analgesics (OTCA) use and use of youth healthcare services among 13–19-year-old adolescents.

**Methods:**

This study was based on national, cross-sectional data from the Ungdata Survey conducted in 2022. The sample was comprised of 16 482 adolescents. Multiple logistic regression was used to analyse the associations between HRQoL, headaches, selected physical symptoms, psychological distress, use of OTCA, PHN availability, sociodemographic variables, and use of the PHN at school or at a YHC. The KIDSCREEN-10 was used to measure HRQoL, and the Hopkins Symptoms Checklist 10 was used to measure symptoms of psychological distress.

**Results:**

Girls used the youth healthcare services more frequently than boys. Better HRQoL was significantly associated with fewer visits to the PHN at school. Girls reported lower HRQoL and mental health, and more pain and frequent OTCA use than boys. When having symptoms of psychological distress, boys had greater odds of visiting the PHN at school than girls. For girls in senior high school, headaches and OTCA use were strongly associated with visiting the PHN at school and the YHC. In senior high school, boys with an immigration background had greater odds of visiting the YHC than native Norwegian boys, while girls with an immigration background were less likely to visit the YHC than native Norwegian girls.

**Conclusions:**

Our results show that more girls than boys use youth healthcare services. When adolescents experience pain, have mental problems, use OTCA, or report low levels of HRQoL, they have greater odds of using youth healthcare services. Youth healthcare services offer excellent opportunities to support and follow up with adolescents. The findings provide important insights into youth healthcare services used by adolescents for various stakeholders, including PHNs and policy makers, with potential implications for future public health efforts.

## Introduction

Healthcare services for youth play a vital role in enhancing adolescent health and well-being [1, 2]. The World Health Organization’s (WHO) [3] guidelines for adolescent-friendly health services highlight systems that are easily accessible and responsive to adolescents’ needs. Their objective is to reach every adolescent without discrimination. However, health services for young people, if existing, are organized heterogeneously across countries [4–8].

In Norway, the school health service and youth health centres (YHCs) are constitutional primary services equipped specifically for all young people. The public health nurse (PHN), who is based at the school health service, is available at all elementary, junior and senior high schools during school hours, while the PHN at the YHC is available in the local community outside of school hours. Each Norwegian municipality organizes the activities these services provide in accordance with its local needs [9]. These youth-friendly health services aim to promote mental and physical health and good social and environmental conditions, prevent diseases and injuries, equalize social inequalities in health, and prevent, uncover and avert violence, abuse and neglect [10]. No referral is needed to access these free-of-charge health services, which in turn provides easy access to the services [9].

Adolescence is a challenging period due to the major change, growth and development that occurs during this time [11]. Further, crucial foundations for future health patterns and health-related quality of life (HRQoL) are established [12]. HRQoL is defined as a multidimensional construct involving an individual’s subjective perspective on their physical, psychological, social and functional aspects of health and well-being [13]. Promoting HRQoL has become a vital goal for health research in recent years [14].

Adolescents’ mental health and general well-being has been shown to decrease throughout adolescence, especially among girls [15, 16]. Gender differences in rates of depression and anxiety levels are generally agreed upon by researchers [17]. Globally, 10–20% of adolescents aged 10–19 years experience mental disorders [18], and the COVID-19 pandemic may have contributed to lower HRQoL, more mental health problems and higher anxiety levels among children and adolescents [19]. Recent studies indicate that the COVID-19 pandemic and its preventive strategies have affected adolescents’ health and well-being, generating, for example, increased loneliness and stress, along with lower life satisfaction and HRQoL [20, 21]. Low HRQoL, in turn, is associated with more mental health problems in children and adolescents [22]. Experiencing pain, with headaches being among the most prevalent self-reported symptoms, is also customary in adolescents, and it has been shown that adolescents who experience pain report significantly lower levels of HRQoL than peers without pain [23–25]. Moreover, previous studies have found an increase in the use of over-the-counter analgesics (OTCA) [26]. A Norwegian study showed that 17.6% of 13–19-year-old Norwegian adolescents had used OTCA weekly [27], with significantly more girls (30.3%) than boys (13.2%) reporting weekly OTCA use. A review by Kiza et al. (2021) revealed that adolescents commonly use OTCA to treat pain, stress and anxiety [28].

Regarding metal illness, one-third of adolescents with severe symptoms of anxiety and depression sought help during the past year, of which only 3.3% sought help from a nurse [29]. Moreover, Potrebny et al. (2021) examined the use of healthcare services between 2014 and 2018 and found that young people with low levels of psychological distress used healthcare services more frequently than peers with high levels of psychological distress [30]. Further, girls have increased contact with health services compared to boys [26, 31–33].

Although our knowledge of health service use among adolescents has increased in recent years, many gaps in knowledge remain to get a more comprehensive picture of the relationship between various interdependent factors that influence the use of healthcare services by adolescents. Based on earlier research and literature we will study the associations between adolescents’ HRQoL, pain, OTCA use and use of youth healthcare services. Examining physical and mental health, HRQoL and OTCA use among adolescents allows for a more complete understanding of adolescents and their use of the service. Such knowledge is crucial in the health promotion work serving adolescents and further, in improvement of youth healthcare services. The aim of this study is to assess possible associations between HRQoL, physical and mental health, OTCA use, PHN availability, immigration background and the use of youth healthcare services in 13–19-year-old adolescents. All analyses will be stratified by gender and school grade level.

## Methods

### Study design and participants

This cross-sectional study was based on data from the Ungdata Survey, a nationally representative study of students from grades 8 to 13 across municipalities in Norway conducted from January to March 2022. During this time, the country and schools were re-opened after the COVID-19 pandemic, which led to significant infection pressure, causing a lower response rate than in 2016 and 2019 [34]. The response rate of the current study was 81% in junior high school and 68% in senior high school. The participants included Norwegian adolescents 13–19 years of age from the County of Agder in Southern Norway [34]. The Ungdata Survey has become an essential source of information on young people’s health and well-being, both at the national and municipal levels [35].

Participating students were not given any incentives. Participating senior high school students in grade 13 were from general academic studies only. Vocational students did not participate. The web-based questionnaire was administered during school hours, with a teacher or an administrator present to answer questions. The adolescents took 30–45 min to complete the questionnaire.

### Measures

The first part of the survey included *sociodemographic information.* Adolescents answered questions about gender, school grade level, home municipality, parents’ birth country and education level.

The *socioeconomic status* (SES) of the participants was measured by asking “Do your parents have university or college education?” with the response options: “no, none of them,” “yes, one of them” and “yes, both of them.”

*HRQoL* was measured using the Norwegian version of the KIDSCREEN-10 self-report questionnaire [36]. The KIDSCREEN-10 is a validated and reliable [37], short, multidimensional measure of generic HRQoL in children and adolescents organized into 10 items: During the past week, have you… “felt fit and well,” “felt full of energy,” “felt sad,” “felt lonely,” “had enough time for yourself,” “been able to do the things that you want to do in your free time,” “parent(s) treated you fairly,” “had fun with your friends,” “got on well at school” and “been able to pay attention.” For each item, five answer categories ranging from “never” to “always” or from “not at all” to “extremely” were provided. The scale for negatively worded items was reversed, and missing values were substituted using the mean of the non-missing items; however, no score was computed if more than one item was left unanswered [36]. The score was then transformed linearly to a 0–100-point scale, with 100 indicating the best HRQoL and 0 the worst. These values were normed to a mean of 50 and a standard deviation of 10 [36]. A clinically meaningful change in HRQoL is 5–10 points [38].

In 2022, the KIDSCREEN-10 questionnaire was a part of the Ungdata Survey for the first time, and it was only administered in the County of Agder.

Symptoms of *psychological distress* were measured using the 10-item Hopkins Symptoms Checklist (HSCL), consisting of two subscales: a depression dimension, which consists of six items and a four-item anxiety dimension [39]. The 10-item version of the HSCL has been shown to perform almost as well as the full version and is recommended for use in both epidemiological and clinical studies to measure psychological distress among adolescents [40, 41]. Participants were asked if they had been affected by any of the following during the past week: “suddenly scared for no reason,” “feeling fearful,” “faintness, dizziness or weakness,” “feeling tense or keyed up,” “blaming yourself for things,” “difficulties in falling asleep or staying asleep,” “feeling blue,” “feelings of worthlessness,” “feeling everything is an effort” and “feeling hopeless about the future.” The ten questions had four response options: (1) “not been affected at all,” (2) “not been affected much,” (3) “been affected quite a lot” and (4) “been affected a great deal.” A mean score of all 10 items was used as a measure of psychological distress. An average score ≥ 1.85 is considered a valid cut-off value for the prediction of psychological distress [42]. Hence, the responses were dichotomized based on this cut-off value to capture the adolescents with symptoms of psychological distress.

Physical health, such as *headache and other physical symptoms*, were measured using the question: “Have you had any of the following symptoms during the past month?”, where responses were given for “headache” and “other physical symptoms (nausea, stomachache, pain in joints, neck or muscles).” The response options were: (1) “never,” (2) “a few times,” (3) “many times” and (4) “daily.”

The adolescents reported *OTCA use* by answering “How often have you used pain relieving medication (Paracetamol, Ibuprofen) during the past month?” The adolescents were then given the response options: “never,” “less than once a week,” “at least weekly,” “many times a week” and “daily.”

The participants were asked about the *use of healthcare services*, and the focus for this study was the PHN at school and the YHCs. The response options to indicate how often the participant used each service during the previous year were as follows: “never,” “one-to-two times,” “three-to-five times” or “six or more times.” For the analysis, these response categories were dichotomized to 0 = did not visit the PHN or YHC, and 1 = visited the PHN or YHC once or more.

*Health service availability* was measured using the question “Is there a PHN at your school?” with the response options: “yes,” “no” and “don’t know.”

*Immigration background* was measured only in senior high school using the question “Where were your parents born?” with the response options: “both were born in Norway,” “one was born in Norway, the other one abroad” and “both were born abroad.” Participants with both parents born abroad were categorized as having immigration backgrounds in further analyses.

### Statistical analyses

All statistical analyses were conducted using IBM SPSS Statistics (version 28). Descriptive statistics were calculated for all variables stratified by gender and presented as counts and percentages for categorical variables and as means with standard deviations (SDs) for continuous variables. Univariate logistic regression was conducted to examine whether HRQoL, headache, selected physical symptoms (nausea, stomachache or pain in neck, muscles or joints), symptoms of psychological distress, OTCA use, PHN availability, school grade level, parents’ educational level and immigration background were associated with the use of youth healthcare services. Variables that were statistically significant (p < 0.05) in these univariate analyses were entered into a multiple regression model. Further multiple logistic regression was used to explore associations between HRQoL, headaches, selected physical symptoms, symptoms of psychological distress, OTCA use, gender, school grade level, immigration background and use of the PHN at school or the use of the YHC during the last 12 months. PHN availability and parents’ education were only included in the multiple regression model with the use of the PHN at school as an outcome. Assumptions for logistic regression were checked and fulfilled. The results are expressed as odds ratios (OR) with 95% confidence intervals (CIs). Analyses were stratified by gender and performed separately for junior and senior high school. A p-value of ≤ 0.05 was required for statistical significance. Sensitivity analyses were performed for participants from junior high school, excluding grade 8 participants, as all grade 8 students in Norway are mandatorily offered a consultation with the PHN at school.

## Results

### Descriptive characteristics of study participants

The sample was comprised of 16 842 adolescents (Table 1), in senior high school, 12.7% of both boys and girls reported having both parents born outside of Norway. A total of n = 5655 (34.3%) adolescents visited the PHN at school during the previous year. Of the included participants, twice as many girls as boys visited the PHN, and three times more girls than boys visited the YHC during the previous 12 months, the figures being 41.6% and 19.3% for the PHN at school, and 15.8% and 5.1% for the YHC, for girls and boys, respectively. Regarding HRQoL, girls reported lower levels than boys. The girls’ mean (SD) for KIDSCREEN-10 was 43.0 (8.3), while the boys reported a mean of 47.6 (9.8). Approximately 1/5 of the boys and half of the girls reported symptoms of psychological distress. More than twice as many girls as boys reported having headaches many times a week or daily during the previous month, with 38.3% of girls and 14.9% of boys reporting such frequency of headaches. Twice as many girls as boys reported having selected physical symptoms many times a week or daily during the previous month, with 40.4% of girls and 19.1% of boys reporting such prevalence of selected physical symptoms. Three times as many girls as boys reported OTCA use many times a week, with 12.4% of girls and 4.3% of boys reporting OTCA use many times a week (Table 1).

**Table 1:** Descriptive characteristics of the variables used in the present study, differentiated by gender. *N* = 16 482

Variables	Levels	Girls *n* = 8380	Boys *n* = 8102
HRQoL			
KIDSCREEN-10	Missing	7428 **(**88.6)	6478 **(**80.0)
		952 (11.4)	1624 (20.0)
KIDSCREEN-10, (0–100), mean (SD)		43.0 (8.3)	47.6 (9.8)
SES			
Parents having education from college or university	No, none of them	1290 (15.4)	1218 (15.0)
	Yes, one of them	2496 (29.8)	2314 (28.6)
	Yes, both	3947 (47.1)	3775 (46.6)
	Missing	647 (7.7)	795 (9.8)
School year			
Grade 8, age 13–14		1462 (17.4)	1572 (19.4)
Grade 9, age 14–15		1418 (16.9)	1447 (17.9)
Grade 10, age 15–16		1402 (16.7)	1407 (17.4)
Grade 11, age 16–17		1462 (17.4)	1438 (17.7)
Grade 12, age 17–18		1304 (15.6)	1246 (15.4)
Grade 13, age 18–19		988 (11.8)	677 (8.4)
Missing		344 (4.2)	315 (3.8)
Ethnicity			
Immigration background^a^	Norwegian born parents	3091 (36.9)	2497 (30.8)
	Foreign born parents	480 (5.7)	428 (5.3)
	Missing	4809 (57.4)	5177 (63.9)
Use of the school health service			
Visited the public health nurse at school the previous 12 months	No	4535 (54.1)	5783 (71.4)
	1–2 times	2191 (26.1)	1190 (14.7)
	3–5 times	746 (8.9)	216 (2.7)
	6 times or more	552 (6.6)	153 (1.9)
	Missing	356 (4.3)	760 (9.3)
Is there a PHN at your school?	Yes	7446 (88.9)	6243 (77.1)
	No	58 (0.7)	86 (1.0)
	Don’t know	270 (3.2)	486 (6.0)
	Missing	606 (7.2)	1287 (15.9)
Use of the youth health centre			
Visited the youth health centre the previous 12 months	No	6620 (79.0)	6882 (85.0)
	1–2 times	974 (11.6)	301 (3.7)
	3–5 times	207 (2.5)	64 (0.8)
	6 times or more	141 (1.7)	52 (0.6)
	Missing	438 (5.2)	803 (9.9)
Pain			
Had headache the last month	Never	741 (8.8)	1574 (19.4)
	A few times	2849 (34.0)	3243 (40.0)
	Many times	2276 (27.2)	1019 (12.6)
	Daily	946 (11.3)	225 (2.3)
	Missing	1568 (18.7)	2041 (25.2)
Had selected physical symptoms (nausea, stomachache, pain in joints, neck or muscles) in the last month	Never	600 (7.2)	1283 (15.8)
	Sometimes	3045 (36.3)	3441 (42.5)
	Many times	2272 (27.1)	1122 (13.8)
	Daily	1118 (13.3)	427 (5.3)
	Missing	1345 (16.1)	1829 (22.6)
Medication use			
OTC analgesics use in the last month	Never	1627 (19.4)	3263 (40.4)
	Less than once a week or at least weakly	5407 (64.5)	3760 (46.4)
	Many times a week or daily	1043 (12.4)	346 (4.3)
	Missing	303 (3.6)	733 (9.0)
Mental illness			
Hopkins Scale Checklist 10	Symptoms of psychological distress	4425 (52.8)	1783 (22.0)
	No symptoms of psychological distress	3202 (38.2)	4859 (60.0)
	Missing	753 (9.0)	1460 (18.0)

Values are number, n (%), if not otherwise stated. ^a^Only asked in senior high school (grades 11–13)

### Univariate associations

#### Use of PHN services at school

In univariate analyses, we tested possible associations between HRQoL, headaches, selected physical symptoms, symptoms of psychological distress, OTCA use, PHN availability, school grade level, parents’ educational level and the use of the PHN at school in junior and senior high school (Table 2). In senior high school, immigration background was also included as a possible predictive factor. Headaches, selected physical symptoms, symptoms of psychological distress and OTCA use were associated with increased odds of visiting the PHN at school, whereas higher levels of HRQoL was associated with decreased odds of visiting the PHN at school (Table 2).

**Table 2:** Univariate associations between HRQoL, physical and mental health, OTCA use, PHN availability, parents’ education, school grade level, immigration background and use of PHN at school in the previous 12 months

	Univariate Analysis				Univariate Analyses			
	Junior High School				Senior High School			
	Girls		Boys		Girls		Boys	
Variables	OR	95% CI	OR	95% CI	OR	95% CI	OR	95% CI
KIDSCREEN-10	**0.96**	**0.96–0.97**	**0.98**	**0.97–0.99**	**0.97**	**0.96–0.98**	**0.97**	**0.95–0.98**
Headache								
• Never	1		1		1		1	
• Sometimes	**1.49**	**1.17–1.89**	**1.45**	**1.19–1.78**	**1.52**	**1.17–1.99**	**1.64**	**1.22–2.21**
• Many times	**2.15**	**1.67–2.75**	**1.76**	**1.37–2.25**	**1.94**	**1.48–2.54**	**2.12**	**1.48–3.03**
• Daily	**2.53**	**1.91–3.34**	**1.83**	**1.18–2.84**	**3.20**	**2.35–4.37**	**2.58**	**1.52–4.37**
Selected physical symptoms^a^								
• Never	1		1		1		1	
• Sometimes	**1.64**	**1.24–2.18**	**1.76**	**1.39–2.24**	**1.48**	**1.12–1.95**	**1.57**	**1.16–2.12**
• Many times	**2.70**	**2.02–3.60**	**2.40**	**1.83–3.16**	**2.03**	**1.53–2.68**	**2.10**	**1.47–2.10**
• Daily	**3.02**	**2.21–4.11**	**2.44**	**1.72–3.45**	**2.69**	**1.98–3.66**	**2.53**	**1.61–3.98**
Symptoms of psychological distress								
• No symptoms	1		1		1		1	
• Symptoms	**1.95**	**1.71–2.23**	**2.04**	**1.73–2.40**	**1.74**	**1.51–2.00**	**2.20**	**1.77–2.74**
OTCA use								
• Never	1		1		1		1	
• Less than once a week	**1.62**	**1.38–1.90**	**1.30**	**1.12–1.51**	**1.62**	**1.34–1.94**	**1.59**	**1.29–1.97**
or at least weakly								
• Many times a week or daily	**2.17**	**1.74–2.70**	**1.77**	**1.29–1.51**	**2.57**	**2.00–3.30**	**2.59**	**1.68–3.99**
PHN at school								
• Yes	1		1		1		1	
• No	0.84	0.41–1.74	1.49	0.80–2.78	**0.25**	**0.09–0.75**	0.17	0.02–1.23
• Don’t know	**0.40**	**0.25–0.65**	**0.56**	**0.35–0.89**	**0.20**	**0.13–0.32**	**0.22**	**0.12–0.38**
School grade level^b^								
• Grade 8 (11)	1		1		1		1	
• Grade 9 (12)	0.96	0.82–1.11	**0.82**	**0.69–0.97**	**1.28**	**1.10–1.49**	1.15	0.92–1.44
• Grade 10 (13)	0.97	0.84–1.13	**0.82**	**0.69–0.98**	1.13	0.95–1.34	1.15	0.88–1.50
Parent’s higher education^c^								
• No	1		1		1		1	
• Yes, one	0.84	0.68–1.04	0.80	0.63–1.02	1.01	0.84–1.22	0.84	0.64–1.11
• Yes, both	**0.74**	**0.61–0.90**	**0.79**	**0.63–0.99**	0.85	0.71–1.02	0.88	0.68–1.15
Immigration background^d^								
• Norwegian born parents					1		1	
• Foreign born parents					0.84	0.69–1.02	**1.35**	**1.02–1.78**

^a^Selected physical symptoms were nausea, stomachache, pain in joints, muscles or neck

^b^Grades 8–10 were junior high school, grades 11–13 were senior high school

^c^Participants were asked: “Do your parents have university or college education?”

^d^Was only asked in senior high school

Values in bold indicate *p*-value < 0.050

Among participants in junior, but not senior high school, having parents who attended college or university was associated with lower odds of visiting the PHN at school, whereas having an immigrant background among boys in senior high school was associated with increased odds of visiting the PHN at school (Table 2).

#### Use of the YHC services

Higher levels of HRQoL and immigration background were associated with lower odds of visiting the YHC, while headaches, selected physical symptoms, symptoms of psychological distress and OTCA use were associated with increased odds (Table 3).

**Table 3:** Univariate associations between HRQoL, physical and mental health, OTC medication use, SES, school grade level, immigration background and use of the youth health centre in the previous 12 months

	Univariate Analyses						Univariate Analyses					
	Junior High School						Senior High School					
	Girls			Boys			Girls			Boys		
Variables	OR	95% CI	*p*-value	OR	95% CI	*p*-value	OR	95% CI	*p*-value	OR	95% CI	*p*-value
KIDSCREEN-10	**0.95**	**0.94–0.97**	**< .001**	**0.96**	**0.95–0.98**	**< .001**	**0.97**	**0.96–0.98**	**< .001**	**0.97**	**0.95–0.99**	**.008**
Headache												
• Never	1			1			1			1		
• Sometimes	1.59	1.00–2.53	.052	1.17	0.79–1.73	.447	**1.71**	**1.20–2.43**	**.003**	**1.76**	**1.07–2.90**	**.026**
• Many times	**2.25**	**1.41–3.59**	**< .001**	**1.95**	**1.25–3.05**	**.003**	**2.45**	**1.73–3.47**	**< .001**	**2.53**	**1.42–4.50**	**.002**
• Daily	**2.52**	**1.53–4.17**	**< .001**	**3.90**	**2.11–7.20**	**< .001**	**3.15**	**2.15–4.62**	**< .001**	**2.93**	**1.30–6.58**	**.009**
Selected physical symptoms^a^												
• Never	1			1			1			1		
• Sometimes	1.22	0.72–2.07	.463	1.00	0.64–1.56	.993	1.36	0.96–1.91	.080	**2.13**	**1.25–3.63**	**.006**
• Many times	**2.53**	**1.50–4.25**	**< .001**	1.91	1.18–3.80	**.008**	**2.03**	**1.44–2.86**	**< .001**	**2.50**	**1.35–4.63**	**.004**
• Daily	**2.82**	**1.64–4.85**	**< .001**	3.25	1.90–5.55	**< .001**	**2.19**	**1.51–3.16**	**< .001**	**3.60**	**1.76–7.37**	**< .001**
Symptoms of psychological distress												
• No symptoms	1			1			1			1		
• Symptoms	**2.49**	**1.97–3.15**	**< .001**	**3.27**	**2.45–4.36**	**< .001**	**1.78**	**1.51–2.11**	**< .001**	**1.54**	**1.08–2.20**	**.017**
OTCA use												
• Never	1			1			1			**1**		
• Less than once a week or at least weekly	**1.64**	**1.22–2.21**	**.001**	1.32	0.98–1.76	.065	**1.93**	**1.52–2.45**	**< .001**	**2.51**	**1.73–3.65**	**< .001**
• Many times a week or daily	**2.73**	**1.91–3.89**	**< .001**	**2.91**	**1.79–4.72**	**< .001**	**3.13**	**2.34–4.20**	**< .001**	**5.19**	**2.85–9.45**	**< .001**
School grade level^b^												
• Grade 8 (11)	1			1			1			1		
• Grade 9 (12)	**1.41**	**1.09–1.83**	**.010**	1.14	0.83–1.56	.431	1.15	0.96–1.37	.145	1.04	0.72–1.50	.842
• Grade 10 (13)	**1.53**	**1.18–1.97**	**.001**	0.98	0.71–1.37	.914	**1.38**	**1.14–1.67**	**.001**	1.44	0.96–2.16	.078
Parent’s higher education^c^												
• No	1			1			1			1		
• Yes, one	0.91	0.65–1.28	.585	0.95	0.61–1.47	.807	1.17	0.94–1.46	.172	1.02	0.66–1.56	0.94
• Yes, both	0.86	0.63–1.17	.331	0.81	0.54–1.23	.332	1.19	0.96–1.47	.114	0.80	0.53–1.23	0.310
Immigration background^d^												
• Norwegian born parents							1			1		
• Foreign born parents							**0.67**	**0.52–0.86**	**.001**	1.49	0.98–2.26	.064

^a^Selected physical symptoms were nausea, stomachache, pain in joints, muscles or neck

^b^Grades 8–10 were junior high school, grades 11–13 were senior high school

^c^Participants were asked: “Do your parents have university or college education?”

^d^Was only asked in senior high school

### Multivariate associations

#### Use of PHN services at school

There was no statistically significant association between parents’ education level and visiting the PHN at school, when adjusting for HRQoL, headaches, selected physical symptoms, OTCA use, PHN availability and school grade level, among junior high school participants. For girls in junior high school and boys in senior high school, higher HRQoL was associated with decreased odds of visiting the PHN at school (Table 4). When HRQoL increased by 10 points (i.e., clinically meaningful change in HRQoL [38]]), the odds of visiting the PHN at school decreased by 18% for both girls (OR = 0.82, 95% CI [0.74–0.90]) and boys (OR = 0.82, 95% CI [0.66–0.90]).

Junior high school students with selected physical symptoms, such as nausea, stomachache or pain in joints, muscles or neck, and senior high school girls with headaches were associated with increased odds of visiting the PHN at school (Table 4). Having selected physical symptoms daily was associated with doubling of the odds of visiting the PHN at school for both girls (OR = 2.03, 95% CI [1.34–3.8]) and boys (OR = 2.02, 95% CI [1.29–3.17]), compared to the junior high school students without selected physical symptoms. Having daily headaches was associated with doubling of (OR = 2.01, 95% CI [1.37–2.95]) the odds of visiting the PHN at school for girls in senior high school, compared to girls without headaches. However, this association was not revealed for boys.

**Table 4:** Multiple logistic regression. associations between KIDSCREEN, physical and mental health, OTC medication use, PHN availability, school grade level, parents’ education level, immigration background and the use of PHN at school in the previous 12 months

	Multivariate Analyses						Multivariate Analyses					
	Junior High School						Senior High School					
	Girls			Boys			Girls			Boys		
Variables	OR	95% CI	*p*-value	OR	95% CI	*p*-value	OR	95% CI	*p*-value	OR	95% CI	*p*-value
KIDSCREEN-10	**0.98**	**0.97–0.99**	**.004**	0.99	0.98–1.00	.077	0.99	0.97–1.00	0.074	**0.98**	**0.96–0.99**	**.007**
Headache												
• Never	1			1			1			1		
• Sometimes	1.02	0.76–1.36	.902	1.15	0.89–1.49	.279	**1.40**	**1.03–1.89**	**.031**	1.05	0.73–1.51	.783
• Many times	1.00	0.73–1.38	.995	1.07	0.76–1.51	.692	1.37	1.00–1.90	.053	0.95	0.59–1.53	.837
• Daily	0.99	0.68–1.45	.975	0.91	0.51–1.64	.753	**2.01**	**1.37–2.95**	**< .001**	1.10	0.55–2.21	.785
Selected physical symptoms^a^												
• Never	1			1			1			1		
• Sometimes	**1.55**	**1.09–2.19**	**.015**	**1.58**	**1.17–2.13**	**.003**	1.06	0.77–1.45	.722	1.31	0.90–1.90	.163
• Many times	**1.96**	**1.35–2.84**	**< .001**	**1.81**	**1.26–2.58**	**.001**	1.25	0.89–1.75	.192	1.47	0.92–2.34	.110
• Daily	**2.03**	**1.34–3.08**	**< .001**	**2.02**	**1.29–3.17**	**.002**	1.40	0.96–2.05	.082	1.48	0.79–2.78	.219
Symptoms of psychological distress												
• No symptoms	1			1			**1**			1		
• Symptoms	**1.40**	**1.14–1.72**	**.001**	**1.72**	**1.35–2.18**	**< .001**	**1.24**	**1.01–1.52**	**.038**	**1.45**	**1.06–1.99**	**.020**
OTCA use												
• Never	1			1			1			1		
• Less than once a week or at least weakly	**1.48**	**1.19–1.82**	**< .001**	1.05	0.85–1.29	.643	**1.31**	**1.05–1.65**	**.020**	**1.47**	**1.10–1.97**	**.009**
• Many times a week or daily	**1.70**	**1.25–2.31**	**< .001**	1.10	0.70–1.74	.678	**1.63**	**1.17–2.27**	**.004**	1.36	0.70–2.66	.366
PHN at school												
• Yes	1			1			1			1		
• No	1.11	0.42–2.98	.833	1.54	0.60–3.97	.368	0.37	0.12–1.12	.078	0.21	0.03–1.62	.134
• Don’t know	**0.35**	**0.19–0.64**	**< .001**	**0.49**	**0.27–0.91**	**.024**	**0.17**	**1.00–0.29**	**< .001**	**0.09**	**0.03–0.23**	**< .001**
School grade level^b^												
• Grade 8 (11)	1			1			1			1		
• Grade 9 (12)	0.83	0.68–1.01	.068	0.85	0.68–1.06	.146	**1.31**	**1.08–1.57**	**.005**	1.06	0.79–1.43	.688
• Grade 10 (13)	**0.80**	**0.66–0.98**	**.027**	0.83	0.66–1.04	.106	1.13	0.93–1.38	.232	1.21	0.87–1.68	.263
Parent’s higher education^c^												
• No	1			1								
• Yes, one	0.91	0.71–1.18	.494	0.77	0.57–1.04	.093						
• Yes, both	0.86	0.68–1.10	.233	0.82	0.62–1.09	.178						
Immigration background^d^												
• Norwegian born parents							1			1		
• Foreign born parents							0.87	0.68–1.12	.290	1.38	0.97–1.98	.075

^a^Selected physical symptoms were nausea, stomachache, pain in joints, muscles or neck

^b^Grades 8–10 were junior high school, grades 11–13 were senior high school

^c^Participants were asked: “Do your parents have university or college education?”

^d^Was only asked in senior high school

Having symptoms of psychological distress was associated with increased odds of visiting the PHN at school for all students, but more so for boys. According to our model, when having such symptoms, junior high school boys were 72% (OR = 1.72, 95% CI [1.35–2.18]) more likely to visit the PHN compared to boys without symptoms, while boys in senior high school were 45% (OR = 1.45, 95% CI [1.06–1.99]) more likely. The comparable figures were 40% (OR = 1.40, 95% CI [1.14–1.72]) for girls in junior high school and 24% (OR = 1.24, 95% CI [1.01–1.52]) for girls in senior high school (Table 4).

Among the female participants, OTCA use was strongly associated with visiting the PHN at school. Likewise, being a boy attending senior high school who used OTCA was associated with increased odds of visiting the PHN. OTCA use of less than once a week or at least weekly was associated with increased odds of visiting the PHN at school by 48% (OR = 1.48, 95% CI [1.19–1.82]) for junior high school girls and 31% (OR = 1.31, 95% CI [1.05–1.65]) for girls in senior high school, compared to girls who never used OTCA (Table 4).

#### Use of YHC services

In multivariate analyses, HRQoL was significantly associated with visits to the YHC. When HRQoL increased by 10 points, the odds of visiting the YHC decreased by 18% (OR = 0.82, 95% CI [0.66–0.95]).

Being a girl in senior high school experiencing headaches was associated with increased odds of visiting the YHC, regardless of headache frequency. Experiencing headaches “sometimes” was associated with increased odds of visiting the YHC by 53% (OR = 1.53, 95% CI [1.03–2.27]), compared to girls without headaches.

Experiencing psychological distress was associated with more than doubling (OR = 2.27, 95% CI [1.48–3.47]) of the odds of visiting the YHC for boys in junior high school, while the figure for girls was 55% (OR = 1.55, 95% CI [1.10–2.18]) (Table 4), compared to students without symptoms.

For all participants in senior high school and girls in junior high school, OTCA use was strongly associated with visiting the YHC. Junior high school girls using OTCA many times a week or daily had 83% (OR = 1.83, 95% CI [1.15–2.92]) increased odds of visiting the YHC, compared to non-users. In senior high school, the association between high use of OTCA and visiting the YHC decreased slightly; however, the association was numerically larger for boys than girls. Girls in senior high school who used OTCA weekly, or less than weekly, were associated with 64% (OR = 1.64, 95% CI [1.23–2.20]) increased odds of visiting the YHC; for boys, the odds were more than double (OR = 2.36, 95% CI [1.44–3.87]) (Table 5), compared to non-users.

**Table 5:** Multiple logistic regression. associations between KIDSCREEN, physical and mental health, OTC medication use, PHN availability, school grade level, parents’ education level, immigration background and the use of the youth health centre in the previous 12 months

	Multivariate Analyses						Multivariate Analyses					
	Junior High School						Senior High School					
	Girls			Boys			Girls			Boys		
Variables	OR	95% CI	*p*-value	OR	95% CI	*p*-value	OR	95% CI	*p*-value	OR	95% CI	*p*-value
KIDSCREEN-10	**0.98**	**0.96–1.00**	**.023**	0.99	0.97–1.01	.308	1.00	0.98–1.01	.604	0.99	0.96–1.01	.293
Headache												
• Never	1			1			1			1		
• Sometimes	1.21	0.72–2.05	.474	1.16	0.69–1.97	.575	**1.53**	**1.03–2.27**	**.037**	1.25	0.67–2.32	.489
• Many times	1.07	0.61–0.87	.812	1.30	0.68–2.47	.423	**1.69**	**1.12–2.55**	**.013**	1.20	0.57–2.56	.632
• Daily	0.74	0.39–1.39	.347	1.77	0.75–4.20	.195	**1.92**	**1.20–3.08**	**.007**	0.81	0.27–2.49	.715
Selected physical symptoms^a^												
• Never	1			1			1			1		
• Sometimes	0.91	0.49–1.66	.750	0.86	0.49–1.52	.599	0.93	0.64–1.37	.726	1.38	0.74–2.59	.313
• Many times	1.48	0.79–2.76	.218	1.08	0.56–2.09	.811	1.17	0.78–1.76	.435	1.46	0.68–3.13	.332
• Daily	1.54	0.79–3.02	.208	1.71	0.82–3.58	.153	1.08	0.69–1.68	.753	1.68	0.65–4.32	.283
Symptoms of psychological distress												
• No symptoms	1			1			1			1		
• Symptoms	**1.55**	**1.10–2.18**	**.012**	**2.27**	**1.48–3.47**	**< .001**	1.19	0.94–1.51	.157	1.15	0.71–1.89	.571
OTCA use												
• Never	1			1			1			1		
• Less than once a week or at least weakly	1.32	0.92–1.90	.133	1.03	0.69–1.53	.894	**1.64**	**1.23–2.20**	**< .001**	**2.36**	**1.44–3.87**	**< .001**
• Many times a week or daily	**1.83**	**1.15–2.92**	**.011**	1.28	0.62–2.64	.500	**2.36**	**1.60–3.46**	**< .001**	**4.37**	**1.84–10.36**	**< .001**
School grade level^b^												
• Grade 8 (11)	1			1			1			1		
• Grade 9 (12)	1.21	0.88–1.66	.241	1.10	0.73–1.67	.651	1.18	0.95–1.47	.143	0.77	0.48–1.24	.278
• Grade 10 (13)	1.31	0.96–1.78	.090	**0.91**	**0.59–1.39**	**< .001**	**1.56**	**1.25–1.95**	**< .001**	1.13	0.69–1.86	.628
Immigration background^c^												
• Norwegian born parents							1			1		
• Foreign born parents							**0.72**	**0.53–0.98**	**.035**	**1.76**	**1.06–2.93**	**.030**

^a^Selected physical symptoms were nausea, stomachache, pain in joints, muscles or neck

^b^Grades 8–10 were junior high school, grades 11–13 were senior high school

^c^Participants were asked: “Do your parents have university or college education?”

^d^Was only asked in senior high school

In senior high school, being a girl with an immigration background was associated with 28% (OR = 0.72, 95% CI [0.53–0.98]) decreased odds of visiting the YHC compared to native Norwegian girls. For boys, the odds were reversed, as boys with immigration backgrounds were associated with 76% (OR = 1.76%, 95% CI [1.06–2.93]) increase in odds to visit the YHC than native Norwegian boys.

#### Sensitivity analysis

All grade 8 students in Norway are offered a consultation with the PHN at school. Hence, sensitivity analysis using the junior high school sample, thereby excluding grade 8 students, was conducted to assess if the associations listed above were present when visiting the PHN at school or visiting the YHC. We found that symptoms of psychological distress in girls were not significantly associated with visiting the PHN at school or the YHC. For all the other presented results, our findings were confirmed also in sensitivity analyses. (Table 6, available upon request).

**Table 6:** Multiple logistic regression in junior high school, excluding grade 8. associations between HRQoL, physical and mental health, OTC medication use, PHN availability, school grade level, and parents’ education level, and the use of PHN at school and the youth health centre in the previous 12 months

	Multivariate Analyses—Outcome: Use of PHN at School						Multivariate Analyses—Outcome: Use of Youth Health Centre					
	Girls			Boys			Girls			Boys		
Variables	OR	95% CI	*p*-value	OR	95% CI	*p*-value	OR	95% CI	*p*-value	OR	95% CI	*p*-value
KIDSCREEN-10	**0.97**	**0.96–0.99**	**.001**	0.99	0.98–1.01	.199	**0.96**	**0.94–0.99**	**.002**	0.98	0.95–1.00	.065
Headache												
• Never	1			1			1			1		
• Sometimes	0.99	0.67–1.44	.971	1.08	0.78–1.51	.633	0.92	0.50–1.68	.782	1.04	0.56–1.96	.897
• Many times	0.90	0.60–1.35	.612	1.09	0.71–1.66	.697	0.89	0.47–1.68	.726	1.23	0.57–2.64	.599
• Daily	0.84	0.53–1.34	.464	0.82	0.38–1.77	.610	0.56	0.27–1.16	.116	0.88	0.27–2.90	.839
Selected physical symptoms												
• Never	1			1			1			1		
• Sometimes	**1.90**	**1.19–3.03**	**.007**	**1.65**	**1.12–2.44**	**.011**	0.95	0.46–1.96	.888	0.84	0.42–1.67	.610
• Many times	**2.93**	**1.79–4.79**	**< .001**	**1.98**	**1.26–3.12**	**.003**	1.59	0.76–3.32	.223	1.10	0.50–2.43	.809
• Daily	**2.59**	**1.51–4.42**	**< .001**	**2.11**	**1.20–3.68**	**.009**	1.35	0.61–3.01	.457	1.51	0.61–3.72	.373
Symptoms of psychological distress												
• No symptoms	1			1			1			1		
• Symptoms of psychological distress	1.23	0.96–1.58	.102	**1.87**	**1.39–2.52**	**< .001**	1.32	0.88–1.96	.177	**2.10**	**1.25–3.52**	**.005**
OTCA												
• Never												
• Less than once a week	1			1			1			1		
or at least weakly	**1.44**	**1.09–1.90**	**.009**	1.03	0.79–1.34	.820	1.29	0.83–2.03	.263	0.84	0.52–1.36	.481
• Many times a week or daily	**1.59**	**1.09–2.33**	**.017**	0.88	0.46–1.65	.680	**2.10**	**1.20–3.64**	**.009**	1.27	0.51–3.17	.616
School grade level												
• Grade 9	1			1			1					
• Grade 10	0.98	0.81–1.18	.806	0.98	0.78–1.24	.875	1.09	0.82–1.45	.552	0.83	0.54–1.27	.388
Is there a PHN at your school?												
• Yes	1			1								
• No	1.16	0.37–3.65	.794	1.79	0.63–5.13	.277						
• Don’t know	**0.45**	**0.23–0.86**	**.017**	**0.34**	**0.15–0.77**	**.010**						
Parent’s education from university or college												
• No	1			1								
• Yes, one	0.95	0.71–1.27	.737	**0.67**	**0.47–0.96**	**.029**						
• Yes, both	0.91	0.69–1.20	.509	0.82	0.59–1.15	.253						

## Discussion

Results from the present cross-sectional study showed that 34.3% of the participating adolescents consulted the PHN at school and that the PHN at school was visited more frequently than the YHC, a trend which corresponds with previous research [30, 31, 43, 44]. These results could be explained by the fact that PHN school services are available at schools, whereas YHC services are located in the adolescents’ home municipality or neighbouring municipality, with after-school opening hours, requiring more effort to visit than the PHN at school.

Another notable finding was that parents’ education was not significantly associated with visiting the youth healthcare services, indicating that the youth healthcare services are a low-threshold service used by adolescents, regardless of SES. This is in line with WHO’s guidelines for adolescent-friendly health services [3] and the national regulation of youth healthcare services [9]. Interestingly, these findings are contrary to a Swedish study in which youth clinic utilization was concentrated among affluent female individuals (aged 16 or older) [45].

We also found that the adolescents reported remarkably lower levels of HRQoL scores than the findings of previous studies of Norwegian adolescents [16, 46]. Similar to our results, other studies have reported a high number of adolescents experiencing mental health problems, especially girls [15, 47, 48]. Hence, low levels of HRQoL and a high prevalence of psychological distress symptoms among adolescents in the present study may not be surprising and could also be related to the potential harmful impact of the COVID-19-pandemic and related measures such as lockdown, etc. [15, 19, 49]. As HRQoL encompasses both physical, psychological, social and functional aspects of well-being [13], mental health problems are expected to have an impact on the HRQoL of individuals. Among Norwegian 13–19-year-old adolescents with mental health problems, a previous study found a stronger inverse association between mental health problems and HRQoL in boys than girls [50]. Our findings also showed a high prevalence of headaches and pain in different sites, which has previously been reported to negatively affect an individual’s HRQoL [22–25]. Further, the fact that adolescence encompasses major growth, development and social role transitions [51] may also influence adolescents’ HRQoL.

To the best of our knowledge, no studies have previously examined possible associations between adolescents’ HRQoL and the use of the PHN at school or the YHC. Results from the present study indicate that adolescents with lower HRQoL used the youth healthcare services more frequently compared to adolescents with higher HRQoL. This is in line with the school healthcare service, YHC regulations and the PHN’s mandate to promote mental and physical health and perform preventive psychosocial work [9]. PHNs at schools and YHCs are in a key position to identify, support and follow up with adolescents in their everyday lives, especially when they experience difficulties.

The gender differences in symptoms of psychological distress observed in the present study conform to previous findings, which have shown lower levels of mental well-being among girls than boys [52]. Adolescents with symptoms of psychological distress were more likely to visit the PHN at school than adolescents without such symptoms. Interestingly, although symptoms of psychological distress were more prevalent in girls, boys with symptoms of psychological distress had greater odds of visiting the PHN at school than girls who had symptoms of psychological distress, both in junior and senior high school. Our findings suggest that boys are more likely to seek help from the PHN when they have mental health challenges. This may represent a shift from the findings of previous studies, in which adolescent boys (age 16–21 and 13–18) did not consult the PHN at school about their mental problems to the same extent as adolescent girls [53, 54].

Among junior high school students, we found that selected physical symptoms, such as stomachache, nausea and pain in joints, muscles or neck, were highly associated with frequency of visits to the PHN at school. A previous study among 17–19 year-olds also found that adolescents with neck and shoulder pain frequently visit the PHN at school [55]. Visiting the PHN at school allows for a unique meeting point and opportunity for in-depth conversation between the PHN and the adolescents. Different physical symptoms may be a gateway to address other difficulties of psychological or physical concern. Regarding visits to the PHN at school for mental health problems, a Norwegian study [53] found that boys (16–21 years) experienced it more easily if their peers thought they visited the PHN for physical concerns. Further, stigma and embarrassment were identified as possible barriers for adolescent boys to seek help for mental health problems, but when overcoming these barriers, they felt positive about their visits and experienced having a new perspective on their problems [53].

Our results confirm the high prevalence of headaches and OTCA use among adolescents in Norway [27, 56, 57], and its positive association with visiting the PHN at school and the YHC, especially among girls. The fact that girls experience more pain than boys is known from previous research [23, 56, 57]. Moreover, previous research has shown that headaches, along with depression and anxiety, are among the strongest predictors for OTCA use [27, 28]. A systematic review by Murray et al. [58] confirmed the high frequency of chronic pain among young adults (aged 15–34), claiming it should be recognized as a major public health concern. Unfortunately, the Ungdata Survey does not distinguish between pain related to menstruation or contraceptive use among girls and other types of pain. Hence, menstruation pain and contraceptive side effects may be confounding factors explaining the high incidence of headaches and selected physical symptoms in girls, as well as symptoms of psychological distress [59–61]. Another study among 15–16-year-old Norwegian adolescents found that frequent users of OTCA experience more pain, sleep fewer hours, have less self-esteem, less academic ambition and more school absenteeism, and engage in binge drinking more frequently than low-frequency OTCA users [62]. Our findings indicate that adolescents who use OTCA are more likely to visit the PHN at school or the YHC than non-users. Hence, the PHNs at school and the YHC should be aware that frequent OTCA use in adolescents can be an expression of physical, psychological and social concerns. Such information should be provided to parents, teachers, PHNs and society as a whole [63].

Our study yielded some interesting findings regarding adolescents with immigration backgrounds. In senior high school, boys with an immigration background were much more likely to visit the YHC than native Norwegian boys. On the contrary, girls with an immigration background visited the YHC less frequently than native Norwegian girls. Information about the use of health services according to ethnicity has not previously been studied in a Norwegian context. However, a recent Swedish study among 16–25-year-olds found lower utilization of YHCs among immigrant youth, with possible barriers being fear of exposure to parents, lack of parental support, communication problems and lack of knowledge about their eligibility for free service [64]. Other barriers hindering migrant youth (16–22) from seeking help for mental problems found in a Canadian study may be previous experiences with authorities not being helpful, lacking trust in healthcare providers, the role of healthcare professionals being unclear and regretting the disclosure after the consultation [65]. Further, an American study pointed out parental reactions, discomfort opening up to others, lack of trust in confidentiality, stigma against mental health services and lack of knowledge thereof as possible barriers [66].

### Strengths and limitations

The present study’s major strengths were the large sample size combined with a high response rate (81% in junior high school and 68% in senior high school). A limitation of this study is the cross-sectional design, which cannot determine causal inference. The associations found between different health issues and the use of youth health services might be influenced by other factors that were not controlled for in the present study. Another limitation is that absent students were not given an opportunity to participate and no information on non-participants was available; thus, we were not able to quantify possible selection bias. However, our results indicate that most participating adolescents come from families with high SES, in terms of parents’ income. Both parents have university or college education in almost half of the sample, which is higher than for people aged 35–59 in the whole population of Agder, as well as Norway as a whole [67, 68]. Thus, the study results may not be representative of the whole population of Norwegian adolescents. Due to social inequalities in health, adolescents with low SES are more likely to develop mental health problems than more affluent peers [69]. Parents’ education level was self-reported by the adolescents, which may have led to some inaccuracies. Junior high school students reported higher numbers (50.8%) of both parents having university or college education than senior high school students (42.3%), possibly indicating an overreporting of educational level by younger students. Further, this study relies on the participants’ self-reported data, which are prone to memory and recall bias, with no medical confirmation. However, the questionnaires used to evaluate HRQoL and mental health are widely used and there is evidence of their validity [40, 70]. The present study did not include questions about visiting a general practitioner or psychologist. Hence, we do not know if our participants sought such help. Therefore, this must be taken into consideration when interpreting our results.

### Public health implications

As PHNs are often available during school hours at all schools, most adolescents have opportunities to receive support and follow-up provided by these services. To enhance adolescents’ mental health and well-being, a recent Norwegian study [52] suggested that it is important to provide information about factors essential to achieving and maintaining good mental health, rather than focusing mainly on factors that cause mental health problems. As previous research has revealed strong associations between physical symptoms and mental health problems [71, 72], health care providers should be aware of the association between pain, low HRQoL, OTCA use and mental health problems and should use targeted interventions to deal with these complex health challenges. Further, the importance of focusing on preventive work in school health services and YHCs has been emphasized by national health authorities [73], as foundations for a healthy life in adulthood are established during adolescence [74]. PHNs have a fundamental role in this important public health work.

## Conclusion

Findings from the present comprehensive study show that low-threshold youth healthcare services, such as youth health services offered at schools and YHCs, are frequently used by adolescents, providing a unique contact point for the PHNs and demonstrating their importance and functioning, in line with current regulations [73]. Although girls use both health services more frequently than boys, boys reporting mental health problems are more likely to visit the PHN at school than girls with mental health problems. Our present study confirms that a large number of adolescents experience pain, have mental health problems, have reduced HRQoL and have high OTCA use [15, 27, 58]. Further, our findings indicate that these vulnerable adolescents seek help from the youth healthcare services, giving the PHNs a genuine opportunity to meet them and provide counselling according to their specific needs and challenges. Our findings emphasize the importance of the youth healthcare services being equipped to work extensively to promote adolescent physical and mental health and HRQoL, as well as applying targeted measures to reach individuals who do not actively pursue the health service themselves. The findings provide important knowledge for PHNs and policy makers, which may have implications for future public health efforts.

## Data Availability

The data and materials from the Ungdata Surveys are closed and stored in a national database administered by NOVA. The data are available for research purposes upon application. For the request of the data, please contact ungdata@oslomet.no. Further information about the study and the questionnaires can be found on the web page https://www.ungdata.no/ (in Norwegian).

## Abbreviations

CI: Confidence interval
GP: General practitioner
HRQoL: Health-related quality of life
HSCL: Hopkins Symptoms Checklist
OR: Odds ratio
OTCA: Over-the-counter analgesics
PHN: Public health nurse
SD: Standard deviation
SES: Socioeconomic status
WHO: World Health Organization
YHC: Youth health centre
